# Acromiohumeral distance and supraspinatus tendon thickness in people with shoulder impingement syndrome compared to asymptomatic age and gender-matched participants: a case control study

**DOI:** 10.1186/s12891-021-04885-3

**Published:** 2021-12-01

**Authors:** Donald J. Hunter, Darren A. Rivett, Sharmaine McKiernan, Suzanne J. Snodgrass

**Affiliations:** Discipline of Physiotherapy, School of Health Sciences, The University of Newcastle, Callaghan, NSW 2308 Australia

**Keywords:** Shoulder impingement syndrome, Subacromial, Supraspinatus, Ultrasound

## Abstract

**Background:**

Shoulder impingement syndrome (SIS) is the most common form of shoulder pain. Conservative and surgical treatments for SIS are often not effective. One such surgical intervention is subacromial decompression, aimed at widening the subacromial space (SAS). A better understanding of the changes in the SAS may help explain the relative ineffectiveness of current interventions.

Objective: To measure the acromiohumeral distance (AHD) and supraspinatus tendon thickness (STT) in people with SIS using a case control study.

**Methods:**

The AHD and STT of 39 participants with SIS ≥3 months and 39 age, gender and dominant arm matched controls were measured using ultrasound imaging. Between-group differences for AHD and STT were compared using t-tests. A linear regression was used to determine if there was a relationship between AHD and STT measures, with group as a covariate.

**Results:**

Compared to controls (mean age 55.7 years, SD 10.6), individuals with SIS (mean age 57.1 years, SD 11.1) had a significantly larger AHD (mean difference 2.14 mm, 95% CI 1.21, 3.07, *p* < 0.001) and STT (mean difference 1.25 mm, 95% CI 0.60, 1.90, *p* < 0.001). The linear regression model indicated an association between AHD and STT (β = 0.59, 95% CI 0.29, 0.89, *p* < 0.01, *R*^2^ = 0.35, *n* = 78), suggesting that as STT increases in size, so does the AHD.

**Conclusion:**

Individuals with SIS had a larger AHD and greater STT than controls. These results suggest the SAS is already wider in people with SIS and that the symptoms associated with SIS may be more related to an increased STT than a smaller SAS.

## Background

Shoulder pain is the third most common musculoskeletal cause of visits to general practitioners [1], with a lifetime prevalence between 8 to 68%, depending on case definition [2]. Shoulder impingement syndrome (SIS) is the most common cause of shoulder pain [3, 4]. SIS is a diagnosis that relates to pain from pathologies within the subacromial space (SAS). Synonymous names for SIS include outlet impingement syndrome [5], subacromial impingement syndrome [6] and subacromial pain syndrome [7]. Pathologies related to SIS include subacromial bursitis, tendonitis of the rotator cuff, partial thickness and/ or full thickness rotator cuff tears and rotator cuff degeneration [8, 9]. Two observational studies [10, 11] and a systematic review [12] of rotator cuff disease report minimal prevalence of SIS in the general population below the age of 40 years, with prevalence consistently increasing with age from 40 [13].

Treatments for SIS range from conservative therapy, usually consisting of manual therapy and/ or exercise, to surgery. However, a recent systematic review concluded that interventions for treating individuals with SIS, whether surgical or conservative, had limited effectiveness [14], with approximately half of all new episodes of shoulder pain continuing to report symptoms beyond 12 months [15]. One of the most common surgical interventions for SIS is subacromial decompression [16]. The use of subacromial decompression in England from 2007 to 2017 increased by 91%, at an estimated cost in excess of one billion pounds [17]. Subacromial decompression is based on the premise that narrowing of the SAS is believed to be associated with the pathologies related to SIS [18–20]. The measurement of the SAS is known as the acromiohumeral distance (AHD), which is the shortest distance between the inferior aspect of the acromion to the closest part of the humeral head [21]. The supraspinatus tendon is the most commonly affected tendon of the rotator cuff in SIS [22, 23] and is located within the SAS.

Previous studies have reported differing findings with regard to the size of the AHD and supraspinatus tendon thickness (STT) in people with SIS as compared to asymptomatic participants. Two studies, using less commonly described methods to measure AHD, reported a smaller AHD in people with SIS than that measured in asymptomatic shoulders [24, 25]. However, three studies, using the accepted and recommended method for measuring AHD [26], found no significant difference in AHD between people with SIS compared to asymptomatic controls. In all three of these studies the AHD was measured using ultrasound (US) imaging devices operated by physical therapists rather than by qualified and experienced sonographers. Similarly, several studies have measured STT in people affected by SIS compared with asymptomatic controls and found the supraspinatus tendon to be thicker in people with SIS [27–29]. Only one study, using overhead athletes in their twenties as participants, reported people with SIS to have a smaller STT measure when compared to asymptomatic controls [30].

Given the presumption of the narrowing of the SAS in SIS as justification for a common decompressive surgery to treat SIS with limited evidence of its effectiveness, further research is necessary to better understand the underlying anatomical pathology associated with SIS. This study investigates whether there is a difference in AHD and STT between individuals with SIS compared to matched, asymptomatic participants. Obtaining AHD and STT measures, using US performed by an experienced sonographer, from both participants with SIS and asymptomatic matched controls may improve our understanding of the underlying pathology of the SAS in people with SIS and lead to more effective treatment outcomes, possibly preventing unnecessary surgery.

## Methods

### Participants

This study was part of a cross-sectional study investigating the possible relationship between thoracic posture (measured from a single lateral radiograph) and SIS [31]. Participants recruited (*n* = 78) were between 40 and 80 years of age, given the increase in prevalence of SIS from the age of forty [10–12]. Thirty-nine asymptomatic individuals with no shoulder symptoms were age, gender and dominant arm matched to 39 participants with SIS. A participant was regarded as asymptomatic if they had not had pain in or around the shoulder while performing daily activities in the previous 3 months, not sought any treatment for shoulder symptoms within the previous 3 months and never had previous shoulder treatments for more than 3 weeks. SIS participants were included if they had experienced shoulder pain for at least 3 months, were positive for at least three out of five orthopaedic clinical tests for SIS (Neer test, Hawkins-Kennedy test, painful arc test, empty can test and the external rotation resistance test) [32], and confirmed as having SIS by a radiologist using US.

Individuals were excluded from either group if they had any condition where undertaking a radiograph was contraindicated (e.g. pregnancy); any history of previous traumatic injury or surgery to the shoulder, neck or back; any known, diagnosed malignancy, infectious disease, or inflammatory disease of the shoulder or spine; or any known, diagnosed neurological conditions (e.g. multiple sclerosis or stroke).

The sample size of this study was determined as the number of participants required for the case-control study investigating differences in posture between individuals with and without SIS [31]. A 5 degree difference in modified Cobb angle between groups (SIS and healthy controls), a standard deviation of 10 degrees (estimated from Katzman et al. (2013) and Fon et al. (1980) [33, 34]) established 34 participants per group to achieve 80% power with a 5% level of significance. Forty participants per group was set as the recruitment target.

Participants were recruited from the community via advertising (flyers and radio websites) and a volunteer research register maintained by a local research institute. Human ethics approval was obtained from the University of Newcastle Human Research Ethics Committee (H-2014-0192) and all participants provided written informed consent before the commencement of this study.

### Participant characteristics

Participants’ age, height (stadiometer: Health-o-meter, Bridgeview, Illinois, USA) and weight (standard analogue scales: A & D, Seven Hills, NSW, Australia) were recorded. Shoulder pain and disability were quantified using the Disabilities of the Arm Shoulder and Hand questionnaire (DASH - minimal detectable change (MDC) 7.9-14.8 points; minimal clinically important difference (MCID) 10.2 points; test-retest reliability, intraclass correlation coefficient (ICC) 0.93-0.98) [35]. The DASH has been shown to be valid and have excellent reliability in patients with SIS [36, 37].

### Ultrasound

The gold standard for diagnosing the pathologies related to SIS is arthroscopic or open surgery [38–40]. However, given surgery is not often required for people with SIS, and with US image quality greatly improved with advancements in technology [41], more recent studies now recommend US as the best imaging option for diagnosing SIS [38, 39, 41–43]. One limitation of US is it is operator dependent. That is, it is desirable that an experienced musculoskeletal sonographer performs the US imaging and an experienced radiologist interpret the findings [43–46]. Given US is non-invasive, has no side effects and can easily and quickly be performed in clinic, US imaging performed by an experienced sonographer and interpreted by a radiologist was selected as the method to be used in the current study for diagnosing the presence of SIS and the measurements of AHD and STT.

SIS was considered to be present if the sonographer observed any evidence of bursitis or any rotator cuff abnormality including tears, tendonitis or degeneration. The diagnosis of SIS could also be dynamic, such as if the sonographer observed signs of complete or partial blocking of humeral head motion, or bunching of the bursa and/ or tendon at the acromion during shoulder abduction [47–50]. The US images were then read by a radiologist experienced in musculoskeletal imaging and blinded to the symptomology of the participant. The radiologist confirmed the presence of SIS for participants suspected of having SIS, and the absence of SIS for asymptomatic participants. All US images were recorded using the Mindray Model M5 (Shenzhen Mindray Bio-medical Electronics Co, Ltd., China) with a 12 MHz linear transducer being employed.

### Acromiohumeral distance (AHD)

**Fig. 1:**
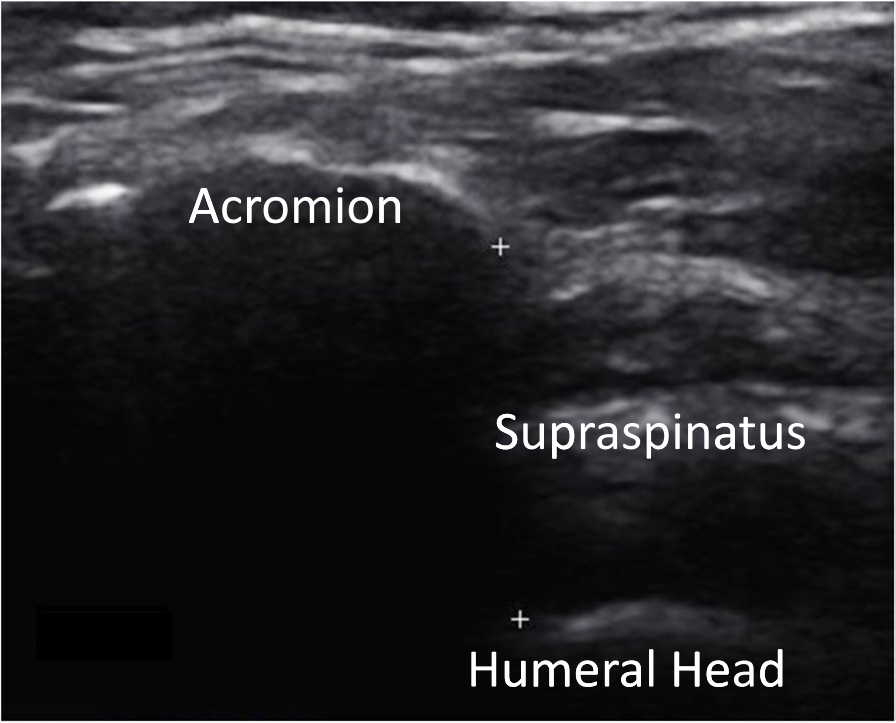
The acromiohumeral distance (AHD, mm) is the distance measured between the two plus signs, indicating the inferior aspect of the acromion and the superior aspect of the humerus

To measure the AHD, participants were seated with the trunk in a neutral position and the arm by their side. The transducer was placed on the anterolateral aspect of the shoulder by the sonographer and the US images recorded (see Fig. 1). The AHD was measured using the US machine’s measurement tool (on-screen ‘callipers’). The sonographer measured the shortest linear distance between the acromion and the humerus by visually locating the inferior aspect of the acromion and the superior aspect of the humerus, marking each with a plus sign as shown in Fig. 1. The US machine automatically calculated the distance between these two points in millimetres (mm). Both SIS and asymptomatic participants had their AHD recorded. This is the most common and best accepted method for measuring AHD with three studies, including a recent systematic review, showing US to have excellent reliability in measuring AHD using this method [26, 45, 51].

### Supraspinatus tendon thickness (STT)

**Fig. 2:**
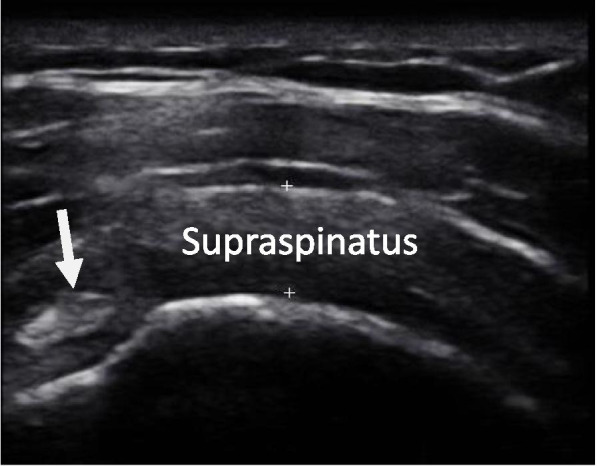
The thickness of the supraspinatus tendon (STT, mm) is measured between the two plus signs, indicating the superior and inferior aspects of the tendon, 10 mm lateral to the long head of biceps tendon (arrow indicates long head of biceps tendon)

To measure STT, participants were seated with the trunk in a neutral position, the elbow flexed to 90 degrees and the shoulder extended as far as comfortable. The transducer was placed on the anterolateral aspect of the shoulder by the sonographer and both the supraspinatus tendon and the long head of biceps tendon were recorded in the same image (see Fig. 2). From the US image the sonographer identified the transverse section of the supraspinatus tendon 10 mm lateral to the edge of the long head of biceps tendon. At this section of the supraspinatus tendon, the STT was calculated using the US machine’s measurement tool. The sonographer visually identified and marked (using plus signs) the superior and inferior aspects of the supraspinatus tendon, as shown in Fig. 2. The US machine automatically calculated the distance between these two points in millimetres (mm). Both SIS and asymptomatic participants had their STT recorded.

### Statistical analysis

Stata statistical software (version 13, College Station, Texas, USA) was used to analyse the data. Descriptive statistics were calculated for participant characteristics (age, gender, weight, height, BMI) and shoulder pain and disability (DASH). T-tests were used to determine if there were any differences in participant characteristics between the SIS and asymptomatic groups despite matching, and whether there were any differences in the AHD and STT between groups. The amount of SAS occupied by the supraspinatus tendon was calculated by the occupation ratio (percentage):

Occupation ratio = STT/AHD *100.

Participant characteristics that differed between groups were included as covariates in linear regression modelling investigating the association between AHD and group, and separately, the association between STT and group. If a difference in descriptive statistics between groups was found, a linear regression model with AHD as the dependent variable, group as an independent variable and the descriptive statistic as a covariate was conducted to assess whether any difference between the groups in AHD could be explained by the descriptive statistic. This was repeated with STT as the dependent variable.

Another regression model was conducted to investigate the linear relationship between AHD and STT (dependent variable = AHD, independent variable = STT), with group as a categorical covariate (group = 0 for asymptomatic controls, group = 1 for SIS, *n* = 78). A further regression model was also used to determine if the magnitude of pain and disability (DASH) was linearly related to STT or AHD for the SIS group (*n* = 39). The model assumptions of linearity and normality of residuals were assessed and met for all models.

Intraclass Correlation Coefficients (ICCs) were calculated for intra-rater and inter-rater reliability of AHD and STT measurements by two raters (an experienced sonographer [rater 1] and an experienced musculoskeletal clinician [rater 2]) on 20 randomly selected participants. The standard error of measurement (SEM) [52] and minimal detectable change (MDC) [53] were also calculated for both the AHD and the STT using data from these 20 participants. With the exception of the first AHD measurement by the sonographer, raters were blinded to the symptomatic status of the participant and to each other’s measurements.

## Results

In the course of confirmation of SIS, or its absence, by the radiologist, there were two participants (one from each group) that did not meet the eligibility criteria. Thus, the presented data are for 78 of the full 80 participants initially recorded. Of the 78 individuals that participated, there were 39 asymptomatic individuals (20 females) and 39 (19 females) with SIS. Age, height, weight, BMI and shoulder disability (DASH) are reported in Table 1. AHD and STT measurements with between-group comparisons are presented in Table 2.

**Table 1:** Characteristics of participants (including mean differences) with shoulder impingement syndrome (SIS, *n* = 39) and age, gender and dominant arm matched asymptomatic control participants (*n* = 39)

Characteristic	All (*n* = 78)	SIS (*n* = 39)	Asymptomatic (*n* = 39)	Mean difference SIS-Asymp (95% CI)	*P* value
Age *(yr)*, mean (SD)	56.4 (10.8)	57.1 (11.1)	55.7 (10.6)	1.3 (−3.6, 6.2)	0.59
Gender *(female)*, number (%)	39 (50)	19 (49)	20 (51)	–	
Weight *(kg)*, mean (SD)	78.4 (16.8)	84.8 (17.6)	71.9 (13.3)	12.9 (6.1, 19.7)	< 0.001
Height *(cm),* mean (SD)	168.4 (8.8)	170.0 (8.0)	166.8 (9.3)	3.3 (−0.7,7.2)	0.10
BMI *(kg/m*^*2*^*)*, mean (SD)	27.5 (4.8)	29.3 (5.3)	25.7 (3.5)	3.6 (1.6, 5.6)	< 0.001
Disability of the Arm, Hand and Shoulder (DASH) questionnaire (/100), mean (SD)	–	26.7 (12.5)	–	–

**Table 2:** Mean (SD) and mean differences (95% CI) between individuals with shoulder impingement syndrome (SIS, *n* = 39) and asymptomatic age, gender and dominant arm matched controls (*n* = 39) for acromiohumeral distance (AHD), supraspinatus tendon thickness (STT) measures and occupation ratio

Measure	Groups		Difference between groups	*P*
	SIS (*n* = 39)	Asymp (*n* = 39)	SIS minus Asymp (95% CI)	
Acromiohumeral distance (mm)	11.97 (2.22)	9.83 (1.88)	2.14 (1.21 to 3.07)	< 0.001
Supraspinatus tendon thickness (mm)	7.11 (1.71)	5.86 (1.13)	1.25 (0.60 to 1.90)	< 0.001
Occupation ratio (%)	60.3 (15.5)	61.4 V(15.6)	−1.0 (−8.0, 6.0)	=0.77

There was no difference in age (years) or height (cm) between groups, though participants with SIS had significantly greater weight (mean difference 12.9 kg, 95% CI 6.1, 18.7, *p* < 0.001) and BMI (3.6 kg/m^2^, 95% CI 1.6, 5.6, < 0.001) than those in the asymptomatic group.

AHD and STT measurements were significantly greater in participants with SIS compared to asymptomatic controls (Table 2). The relationship between AHD and group was further investigated accounting for BMI (rather than weight, as BMI is a function of weight, with height). In the linear regression model examining the association between AHD and group with BMI as a covariate, BMI was significant (β = 0.16, 95% CI 0.06, 0.26, *p* = 0.002) and thus accounted for a portion of the difference in AHD between groups (group: β = 1.58, 95% CI 0.63, 2.52, *p* = 0.001, *R*^2^ = 0.31, *n* = 78; group = 1 for the SIS group and group = 0 for the asymptomatic group).

BMI was also significant (β = 0.16, 95% CI 0.10, 0.23, *p* < 0.001) in the model investigating the relationship between STT and group, accounting for a portion of the difference in STT between groups (group: β = 0.68, 95% CI 0.07, 1.29, *p* = 0.03, *R*^2^ = 0.38, *n* = 78). No other descriptive statistic (height, gender or age) significantly explained the difference in AHD or STT between groups. There was no difference in occupation ratio between groups, even when adjusted for BMI.

There was a positive linear association between AHD and STT (β = 0.59, 95% CI 0.29, 0.89, *p* < 0.001, *R*^2^ = 0.35, *n* = 78), indicating that as the STT increased in size, so did the AHD. In this model, the SIS group (as the categorical independent variable) had an AHD measure of 1.40 mm greater, on average, than the asymptomatic control group (β = 1.40, 95% CI 0.46, 2.32, *p* = 0.004). Adding weight or BMI as covariates did not improve the linear regression model, hence are not included. There was no linear relationship between STT (β = 0.79, 95% CI -1.62, 3.21, *p* = 0.51) or AHD (β = − 0.64, 95% CI -2.49, 1.22, *p* = 0.49) and the DASH score (pain and disability) for the SIS group.

Intra-rater reliability for AHD measurements was excellent for rater 1 (ICC (2,1) = 0.99; 95% CI 0.98, 1.00) and good to excellent for rater 2 (0.85; 0.63, 0.94) [54].. Inter-rater reliability was good to excellent (ICC (2,1) = 0.92; 95% CI 0.80, 0.97). The SEM of the AHD was 0.44 mm with the MDC = 1.22 mm.

Intra-rater reliability for STT measurements was excellent for both raters (rater 1: ICC (2,1) = 0.99; 95% CI 0.98, 1.00; rater 2: 0.98;0.96, 0.99). Inter-rater reliability was excellent (ICC (2,1) = 0.95; 95% CI 0.88, 0.98). The SEM of the STT was 0.33 mm with the MDC = 0.91 mm.

## Discussion

This study set out to compare the AHD and STT between people with SIS and matched controls. The results of this study indicate that participants with SIS had a significantly greater AHD (2.14 mm on average, as measured on US) and STT (1.25 mm) as compared to asymptomatic controls, both measures greater than their respective MDCs. While adjusting for BMI resulted in the AHD and STT between-group differences being smaller, the SIS group still recorded significantly larger average AHD and STT measures than the asymptomatic controls. One proposed mechanism for SIS is a larger occupation ratio (STT/AHD) [27, 55], with the supraspinatus tendon occupying a greater amount of the SAS. However, this study found no difference in occupation ratio between groups.

There was a linear association between AHD and STT, indicating that as the STT increased, so did the AHD. These findings suggest that people with shoulder pain from SIS have a thickened supraspinatus tendon which is associated with an increased distance between the humerus and the acromion. There was no linear relationship between the magnitude of pain and disability an individual with SIS was experiencing and the AHD or thickness of their supraspinatus tendon.

Published studies with similar methodologies to the current study comparing the AHD between SIS participants and asymptomatic controls all found no difference between groups. Desmeules et al. [56] in an underpowered study of seven SIS participants and 13 healthy controls with an average age of 34 years, found no significant difference in the AHD between groups. In the study by Michener et al. [27] with SIS and asymptomatic groups each having an average age of 45 years (20 in each group), no significant difference in the AHD was also reported. Similarly, Navarro-Ledesma and Luque-Suarez [57] also showed no difference in the AHD between groups with an average age of 46 years (76 SIS participants, 40 controls). However, all three studies had participants at least 10 years younger than in the current study and the AHD was measured using US operated by a physical therapist rather than by an experienced sonographer as in the present study.

Conversely, two prior studies investigating AHD in participants with SIS found the AHD to be smaller in SIS participants than controls. Using US, Cholewinski et al. [25] found participants with SIS (*n* = 57, mean age 56 years), when compared to healthy controls (*n* = 36, mean age 57), had a smaller AHD. However, the method used to measure the AHD was different to the current study and its reliability was not reported nor in any previous paper identified. Park et al. [24] found a significantly smaller AHD in participants with SIS compared to asymptomatic controls, but measured the AHD from MRI images using different anatomical landmarks from the current study and with participants lying supine, thus altering the effect of gravity on the articular relationships. Given the US method for measuring AHD has the participant sitting upright, measurements from the current study are therefore not directly comparable with those of Park et al. (2018).

Benitez-Martinez et al. [30] and Leong et al. [28] investigated AHD in overhead athletes, with both investigations finding no difference between participants with SIS compared to asymptomatic controls (mean age 26.1 and 21.5 years respectively). The repetitive overhead nature of the sporting activity and the relatively young age of participants in both studies limits the ability to generalise their findings to the greater population and thus compare results to the present study.

For studies with similar methodologies to the current study and which compared STT between participants with SIS and asymptomatic controls, similar results are generally reported. Michener et al. [27] found that people with SIS (*n* = 20, mean age 45.1 years) had a greater STT compared to matched asymptomatic controls (*n* = 20, mean age 45). Likewise, Joensen et al. [29] also found people with unilateral supraspinatus tendon related pain (*n* = 64, mean age 48 years) had greater STT compared to the asymptomatic shoulder of the same individual. Only one study reported no difference in STT between SIS participants compared to controls [25].

The findings from the current study of greater AHD and STT in individuals with SIS compared to controls, in conjunction with the results of studies [27, 29, 56, 57] using similar methodologies and samples, is potentially at odds with the theorised mechanism that symptomatic pathology related to SIS is due to a narrowing of the SAS [23, 27]. A larger AHD suggests a greater SAS in individuals with SIS compared to controls. Notably, a recent multi-centre randomised surgical trial investigating subacromial decompression surgery, a common procedure to treat symptoms related to SIS by increasing the size of the SAS, reported that this surgery is no better than placebo [16]. Taken together, the findings of this and the current study suggest the painful shoulder symptoms associated with SIS are unlikely to be related to the size of the SAS. Symptoms may instead be related to a thickening of the supraspinatus tendon, as observed in the present study and also those of Michener et al. (2015) and Joensen et al. (2009). This pathoanatomical evidence is likely the result of degeneration and chronic overuse of the supraspinatus tendon [12, 58], suggesting management of patients with probable SIS should incorporate treatment directed at reducing the thickening of the supraspinatus tendon [59–61] and educating patients on avoiding behaviour that irritates the tendon. While further research is needed, this approach may promise more relief for people suffering the symptoms of SIS than surgical treatment addressing the size of the SAS.

### Limitations

This was a cross-sectional study investigating AHD and STT in people with SIS at one point in time, thus only associations between study factors can be demonstrated. While it appears that a greater AHD or greater STT may be risk factors for SIS, given this is a cross-sectional case-control study, it is not possible to infer causality. A large longitudinal study following, initially younger, people over several decades would be required to irrefutably demonstrate whether increases in AHD or STT increase the risk of developing SIS.

The measures of AHD and STT were recorded with a participant’s arm by their side. Given that pain associated with SIS can occur with various movements of the arm, the recording of AHD and STT measures at varying degrees of shoulder flexion and abduction may provide more information as to the cause of the pain associated with SIS. Also, when the US images were recorded, the sonographer was not blinded to the symptomatology of the participant. However, the radiologist reading the images was blinded to symptomatic status.

Another limitation is that our results can only be generalised to people aged 40 years or older. For this study we purposefully restricted the inclusion of participants to 40 years or older, given the minimal prevalence of rotator cuff disease in people under 40 years of age [10–12]. If we had included participants in their 20s, then it is likely the aetiology of their SIS would have been different to the participants of the current study [20, 62]. More research is required using similar methodologies to the current study, employing participants of different ages to the current study but with likely similar SIS aetiologies.

A final limitation was that it was not feasible to match participants in each group for BMI, and BMI was associated with the outcomes. We accounted for between-group differences in BMI using regression modelling, but future studies may consider matching for BMI in addition to age and gender.

## Conclusion

This study found greater AHD and STT measures in people with SIS compared to matched, asymptomatic controls, and that these two measures are linearly associated. This suggests that symptoms consistent with SIS may be related to a larger SAS with thickening of the supraspinatus tendon, rather than a narrowing of the SAS.

## Data Availability

The datasets used and/ or analysed during the current study are available from the corresponding author on reasonable request.

## Abbreviations

SIS: Shoulder impingement syndrome
SAS: Subacromial space
AHD: Acromiohumeral distance
STT: Supraspinatus tendon thickness
US: Ultrasound
DASH: Disabilities of the Arm Shoulder and Hand

## Declarations

This research was conducted in accordance with the Declaration of Helsinki**.**
